# The impact of retrieval suppression on conceptual implicit memory

**DOI:** 10.1080/09658211.2018.1554079

**Published:** 2018-12-07

**Authors:** Assaf Taubenfeld, Michael C. Anderson, Daniel A. Levy

**Affiliations:** a Baruch Ivcher School of Psychology, Interdisciplinary Center Herzliya, Herzliya, Israel; b MRC Cognition and Brain Sciences Unit, University of Cambridge, Cambridge, United Kingdom

**Keywords:** Suppression, inhibition, implicit memory, priming, retrieval

## Abstract

When people suppress retrieval of episodic memories, it can induce forgetting on later direct tests of memory for those events. Recent reports indicate that suppressing retrieval affects less conscious, unintentional retrieval of unwanted memories as well, at least on perceptually-oriented indirect tests. In the current study we examined how suppressing retrieval affects conceptual implicit memory for the suppressed content, using a category verification task. Participants studied cue-target words pairs in which the targets were exemplars of 22 semantic categories, such as vegetables or occupations. They then repeatedly retrieved or suppressed the targets in response to the cues for some of those pairs. Afterwards, they were exposed to the targets intermixed with novel items, one at a time, and asked to verify the membership of each of the words in a semantic category, as quickly as possible. Judgment response times to studied words were faster than to unstudied exemplars, reflecting repetition priming, as has been previously observed. Strikingly, the beneficial effects of prior exposure on response time were eliminated for targets that had been suppressed. Follow-up explicit memory tests also demonstrated that retrieval suppression continued to disrupt episodic recall for the items that had been just been re-exposed on the category verification test. These findings support the contention that the effects of retrieval suppression are not limited to episodic memory, but also affect indirect expressions of those memories on conceptually oriented tests, raising the possibility that underlying semantic representations of suppressed content are affected.

The human capacity to monitor and control the accessibility of memory traces is an important factor mediating how our past experiences affect our thoughts and emotions. One avenue for understanding the cognitive processes and neural mechanisms underlying this faculty is provided by investigating retrieval suppression – the ability to stop the retrieval of memories into consciousness via inhibitory control. A corpus of work (e.g., Anderson & Green, 2001; Anderson & Hanslmayr, 2014; B. Levy & Anderson, 2008) has demonstrated that similarly to our ability to stop physical actions, people also can stop the retrieval process. To study this retrieval stopping process, the Think/No-Think paradigm (Anderson & Green, 2001) was modelled on the Go/No-go task, in which people are asked to withhold a prepotent motor response whenever a certain cue appears. Similarly, in the Think/No-Think paradigm participants receive reminders of previously encoded memories and are asked to stop a cognitive function – in this case, their memory retrieval – through intentional inhibition of memory traces. Participants first study cue-target pairs (e.g., ordeal – roach) and are trained to recall the target word (roach) when they see the cue (ordeal). In the next stage, the cue word is presented, and participants are asked to either retrieve the target word (the “Think” condition), or to suppress its retrieval (the “No-Think” condition). After that Think/No-Think phase, participants perform retrieval tasks to evaluate how the prior suppression trials affected their memory for the suppressed content (Anderson & Huddleston, 2012). Typically, repeatedly suppressing No-Think items impairs their later recallability, a phenomenon known as suppression-induced forgetting. Suppression-induced forgetting indexes the lingering effects of retrieval suppression on the unwanted content, and has often been taken to reflect the impact of an underlying inhibitory control process (Anderson & Green, 2001; Anderson & Hanslmayr, 2014).

Two types of memory tests are usually used in the final test phase of the Think/No-Think paradigm to measure suppression-induced forgetting. One is a “Same Probe” cued recall test, in which participants receive the original cue words (e.g., “ordeal” in the preceding example) and are asked to recall the words studied with them (e.g., “roach”). The other is an “Independent Probe” test, in which a novel extralist semantic cue (often a category) is presented along with a letter stem of the target (e.g., “insect- r____”), and participants are asked to recall the studied word that is a member of that category and begins with the designated letter (Anderson & Huddleston, 2012). Many studies have demonstrated that suppression-induced forgetting occurs on both types of test, which is often taken to indicate that suppression-induced forgetting is cue-independent, consistent with the involvement of an inhibitory process in causing this effect (see, e.g., Anderson & Green, 2001; Anderson & Hanslmayr, 2014; Anderson & Spellman, 1995).

A core claim of the inhibitory control account of suppression-induced forgetting is that suppression decreases the accessibility of the unwanted memory trace itself by an active inhibition process (Anderson & Green, 2001). Although reduced recall on the two tests for suppressed items is broadly consistent with this hypothesis, forgetting on the Same Probe test can also be produced by interference effects that are specific to the Same Probe cue (Anderson & Green, 2001). Indeed, several studies point to an additional contribution of non-inhibitory interference effects to suppression-induced forgetting on the Same Probe test (e.g., Noreen & de Fockert, 2017; Wang, Cao, Zhu, Cai, & Wu, 2015).

To isolate the contributions of inhibition, researchers have applied the Independent Probe test to assess generalised suppression of the memory trace. For example, after initial study of a pair such as “cage-banana” and then suppression of retrieval (“banana”) to the cue (“cage”) (a No-Think trial), a follow-up assessment of that manipulation reveals that the recall of “banana” is impaired not only when it is tested with the studied cue (e.g., “cage”), but also when it is tested with a new, independent cue (e.g., “monkey-b_____”). Diminished retrieval success for No-Think items during the Independent Probe test supports the assertion that the memory trace of the suppressed response is weakened, rather than suppression affecting retrieval of the specific associative pathway (Anderson & B. Levy, 2009). The format of the Independent Probe test additionally addresses the potential for demand characteristics, because that format is different than the one to which the participant adhered during the suppression trials of the procedure, ostensibly enabling him or her to respond using the full strength of whatever memory representation survives suppression. To the extent that there is still relative retrieval failure, this is taken to be an indication of weakening of the episodic memory trace of the originally studied item (see Anderson, 2003, for an overview of best practices on using the independent probe method to infer inhibition; see Discussion for further commentary).

Further evidence for the claim that retrieval suppression acts directly on previously formed memory representations may be provided by examining retrieval inhibition effects on implicit expressions of memory, such as priming. Priming represents the unintended influence of past experience on current performance or behaviour (Schacter & Buckner, 1998), manifest in changes in a person’s ability to identify, process, or produce an item as a result of a specific prior encounter (Schacter, 1987). Kim and Yi (2013) used the Think/No-Think paradigm to test whether the forgetting effect caused by retrieval suppression also extends to the perceptual priming domain. Participants studied pairs composed of word cues and object line drawing targets and then engaged in a standard Think/No-Think task. Kim and Yi replaced the final cued-recall task of the Think/No-Think paradigm with a perceptual identification task conducted on drawings of the objects. In a series of three experiments, the researchers found that the suppression procedure significantly weakened subsequent perceptual priming benefits in the identification of the objects. Gagnepain, Henson, and Anderson (2014) used a similar procedure, but with photographs of objects, and reported that the behavioural effects of suppression in reducing priming were accompanied by reduced activity in neocortical areas involved in perceiving objects. It should be noted, though, that Angello, Storm, and Smith (2015) have reported that in indirect tests of memory for visual form (e.g., orthography) evidence of suppression was not obtained even though explicit versions of the same task yielded evidence of suppression-induced forgetting.

Alongside stimulus-specific perceptual priming, the impact of conceptual information inherent in experienced stimuli on subsequent thought or behaviour may be expressed as conceptual priming. The effect of suppression on this conceptual form of priming is particularly interesting because reducing indirect conceptual influences of an event might be beneficial to one’s efforts to controlling unwanted memories or thoughts. The effects of retrieval suppression on conceptual priming have been studied by Hertel, Large, Stück, and A. Levy (2012), who report that suppression practice led to diminished subsequent production of suppressed targets in a free-association procedure. Furthermore, when cues related to suppressed targets were homographic, other associates related to the meaning of suppressed targets were also produced below-baseline (but only when suppression was aided by thought substitution). Hertel and colleagues suggest that using free association cues reduces contributions of explicit memory. If so, their findings could be interpreted as an effect of retrieval suppression on the underlying semantic representation of the to-be-suppressed stimulus. Hertel et al. did not, however, examine suppression-induced forgetting with a direct suppression instruction in which participants are instructed to avoid generating thought substitutes in response to No-Think cues (Benoit & Anderson, 2012; Bergström, de Fockert, & Richardson-Klavehn, 2009); rather, participants either were asked to retrieve distracting substitute thoughts, or given no special suppression-instructions. Because their evidence for suppression-induced forgetting was limited to cases in which thought substitutes were given, the effect may reflect associative interference from the thought substitutes rather than inhibition. To address whether inhibition truly reduces conceptual priming, it seems desirable to investigate whether suppression-induced forgetting arises in other conceptual implicit memory tests that are not as subject to associative interference accounts, preferably with direct suppression instructions.

We have previously demonstrated that conceptual priming may be entirely independent of declarative memory (Levy, Stark, & Squire, 2004). In that study, we examined two kinds of semantic priming – the effects of prior exposure to words on their subsequent production in a semantic free association task (similar to the task of Hertel and colleagues), and the impact of prior study on the speed of category verification. In the latter paradigm, we conducted encoding of a mixed list of words that were uncommon exemplars of a range of categories, such as fruit, metals, and occupations. Following a delay, participants were presented with queries such as: “Is *quince* a type of *fruit*?”, or “Is *pewter* a type of *vehicle*?”, which they were asked to answer yes or no as fast as possible by keypresses. Some of terms queried had been studied previously, and some not. Conceptual priming was expressed in faster responses to the studied words (a 160 ms advantage for studied materials), for both yes and no answers. Importantly, amnesic patients exhibited the same degree of priming as age- and education-matched healthy control participants. Even amnesic patients with extensive medial temporal lobe lesions, who at the same five-minute delay performed at chance in tests of recognition memory for parallel words, exhibited the priming benefits. Those findings converge with previous work regarding perceptual priming indicating that such forms of memory are doubly dissociated from explicit declarative memory (Gabrieli, Fleischman, Keane, Reminger, & Morrell, 1995; Hamann & Squire, 1997).

In healthy participants, response time advantages for studied stimuli in conceptual processing such as the category verification task (CVT) may reflect a combination of implicit priming and explicit declarative memory for the previously studied stimuli. However, the CVT paradigm requires no reference to the study episode, and is likely performed as an independent process without strategic utilisation of explicit memory (as suggested by the identical priming response time benefits in amnesic patients and healthy participants in that task in Levy et al., 2004). Accordingly, conceptual priming such as evidenced in the CVT may provide an effective measure of the ability of retrieval suppression to weaken a mental representation that had been previously strengthened by episodic encoding. The indirect nature of the task, requiring no reference at all to the encoding episode and no production component, likely reduces the involvement of explicit retrieval processes, revealing the effect of suppression on conceptual representations. Finally, the absence of the originally studied reminder cue from the paired associations during the category verification task makes an interpretation in terms of interference processes less likely, especially when combined with direct suppression instructions designed to minimise the encoding of alternative associations (Benoit & Anderson, 2012; Bergström et al., 2009).

In the present study, we used the Think/No-Think paradigm’s first phase of associative learning as the priming phase. To test the hypothesis that memory suppression affects implicit memory, we measured response time differences in the category verification task for category exemplars assigned to the three conditions created in the Think/No-Think procedure: only studied (e.g., Baseline items), studied and retrieved (e.g., Think items), studied and suppressed (e.g., No-Think items), as well as in a fourth condition – never studied (e.g., Unprimed baseline). We hypothesised that retrieval suppression would affect not only standard Same Probe and Independent Probe tests of explicit memory, but conceptual implicit memory as well.

## Method

### Participants

40 undergraduate students (30 females; mean age = 24, SD = 1.9; range 21–28) were recruited to participate in exchange for payment and/or academic requirement credit (at the rate of 40 shekels [about 10 Euros] per hour). To maximise motivation in performing the Think/No-think procedure, participants were advised that they would be participating in a study of attentional control conducted in the framework of a research project to promote neuropsychological rehabilitation (which is indeed the main programme of research conducted in the laboratory). Informed consent was obtained from all participants for a protocol approved by the Institutional Review Board of the Interdisciplinary Center Herzliya.

### Materials

Four uncommon exemplars were selected for 22 categories of objects, using norms determined for Hebrew usage (Henik, Rubinsten, & Anaki, 2005). For example, for the category “mammals”, the exemplars were “zebra”, “panther”, “kangaroo” and “fox”. For each exemplar, one weak associate, based on norms determined for Hebrew usage, was chosen as a cue enabling the construction of a meaningful association for initial study, cued recall testing, and the Think/No-Think procedure. A second weak associate was chosen for use as an independent probe in later phase of the experiment. For example, for “zebra”, one associate was “safari” and another was “hoof”, while for “fox”, one associate was “forest” and another was “tail”. Thus, a total of 88 critical triplets were prepared. These 88 sets were assigned, in counterbalanced fashion across participants, to one of four conditions: Initial Study-Test with subsequent retrieval practice (Think condition); Initial Study-Test with subsequent suppression practice (No-Think condition); Initial Study-Test without subsequent practice (Study-only condition); No Initial Study-Test (Category verification task Test-only baseline condition). English translation of all the categories and exemplar names is provided in Appendix 1. An additional 14 unrelated word pairs were constructed for use as fillers and practice trials.

### Procedure

#### Associative learning

**Figure 1.**
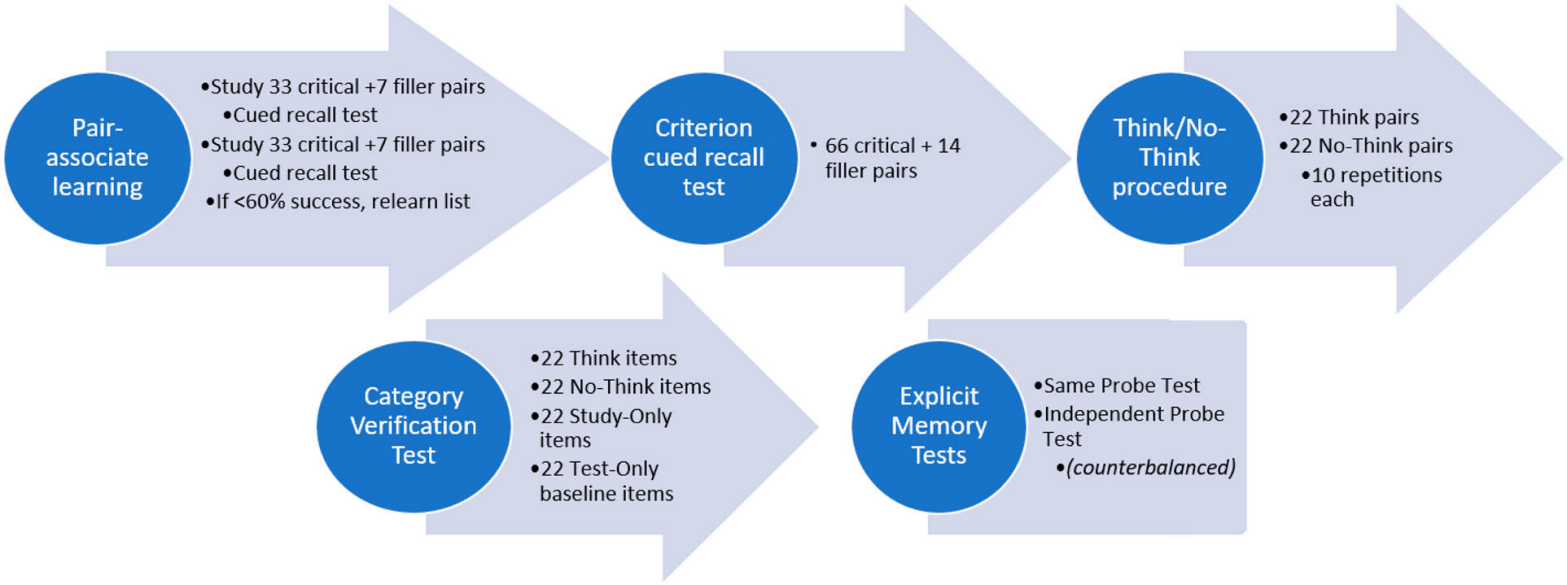
Schematic diagram of experimental procedures.

For an overview of the experimental procedures, see Figure 1. In the first stage of the experiment, each participant was trained on 66 critical and 14 filler word pairs, for a total of 80 study pairs. These were divided into two lists, studied separately, each including 33 critical and 7 filler word pairs, with filler pairs at the beginning and at the end of the lists and 3 additional fillers randomly distributed among the critical pairs. The word pairs were presented individually for 5 s in the centre of a computer screen (black font on grey background), with the cue word displayed to the right of the target (as Hebrew is read right-to-left). Participants were instructed to attempt to think of an association between the two presented words, in preparation for a later unspecified test. Trials were separated by a 1 s blank-screen interval. After learning the first list, participants were probed with cue words from that list, and asked to recall corresponding target words and to say them aloud as quickly as possible. The correct answer was presented onscreen for 2.5 s, either immediately after the participant’s verbal response, or 5 s after cue presentation if there was no response. The test phase both started and ended with two fillers. The same procedure was then followed for the second list. Participants completed either two study-test cycles of both lists, or one cycle for both lists if a minimum of 60% of the targets were correctly recalled in the initial cycle. After the study-test learning phase, participants were again tested on all 66 critical pairs and 14 filler word pairs.

#### Think/no-think procedure

After completing the study phase, subjects were given the Think/No-Think phase instructions. This started with demonstration and practice trials conducted on the 14 filler word pairs. 7 word pairs were assigned to the suppression condition, and 7 word pairs assigned to the response condition. After the practice phase, participants were asked to answer a diagnostic questionnaire to ensure they understood the instructions and followed them closely. Participants were given a 5-minute break after answering the questionnaire before the actual Think/No-Think phase. After the break, instructions were read again as a refresher. This procedure was then conducted for 44 critical pairs, of which 22 were assigned to the Think (respond) condition or the No-Think (suppress) condition. These pairs were each presented 10 times during across 4 blocks (i.e., each pair appeared 2–3 times per block), with each block lasting approximately 7 min. Each block started with 2 fillers, followed by 110 cue words from the studied pairs, with Think and No-Think trials randomly intermixed. In the Think condition (indicated by a green cue word), participants were instructed to think of the target word when the cue word appeared. In the No-Think condition (indicated by a red cue word), participants were instructed not to think of the target word when the cue word appeared, and to prevent it from entering their consciousness. Specifically, we employed Direct Suppression instructions (Benoit & Anderson, 2012), which stressed to participants that they were to suppress retrieval, while also not generating distracting thoughts. Cues were presented for 3 s in the centre of the screen. Trials were separated by a 500-ms blank-screen interval. After each block, participants were asked to answer a short questionnaire about their ability to recall or suppress responses as required, in order to maintain their level of concentration. A 5 min rest break followed this stage.

#### Category verification task

After the Think/No-Think phase and rest break, subjects were given the Category Verification task instructions. Participants were asked to answer yes/no by key press (using the index and middle fingers of their dominant hand) to questions about category membership of target item names (e.g., “Is **fig** a kind of **vehicle**?”; “Is **cider** a kind of **beverage**?”). The target item and category names were presented in **boldface**. 88 target names were presented (22 from Think pairs, 22 from No-Think pairs, 22 from study-phase-only pairs and 22 completely new words). This task started and ended with 2 fillers. For the 22 categories from which 4 words each were divided among the experimental conditions, 2 category members were presented in questions that were to be answered “Yes” and 2 were presented in questions that were to be answered “No”. Category names for questions that were to be answered “No” were different from those providing the exemplars for experimental conditions. Assignment of item names to Yes/No responses was counterbalanced across participants. The question was displayed in the centre of the screen until response; the critical dependent measure for this task was response time. Trials in which category verification decisions were incorrect were removed from response-time analyses; the mean number of such removals was 4.4, SD = .04. A 1 min break was given before moving to the next test.

#### Same-probe and independent-probe tests

After the Category Verification task, participants had a short recall practice for both Same Probe and Independent Probe tests. 14 filler word pairs were used in order to make sure they understood the instructions and did not confuse between the two tests. After this practice, participants completed the Same Probe and Independent Probe tests, each for all 66 words from the associative learning phase, in an order counterbalanced across participants. The Same Probe test cued participants with the original hint word (e.g., for the target word “owl”, the original cue word was “air”), whereas the Independent Probe test cued them with a related hint word and the first letter of the item (e.g., “night - o_____”). Participants were given 5 s to recall each item, and were instructed to think of the response that fit each cue and say it aloud, regardless of the colour in which the cue word had been presented in the Think/No-Think phase.

Stimuli were presented and dependent measures collected using E-Prime 2.0 software (PST, Pittsburgh). In the category verification task, trials in which participants’ RT was 3 SDs above each participant’s mean RT were removed from analyses. Statistical analyses were conducted with SPSS 21 (IBM, Armonk). We followed-up null results with Bayesian analyses, performed using R version 3.4.1 as implemented by RStudio version 1.0153, with a BayesFactor package (Morey & Rouder, 2015).

## Results

Our initial analyses focused on the effects of retrieval suppression as expressed in measures employed in earlier studies – recall success in Same Probe and Independent Probe tests. We report an analysis that considers all of the studied items (Unconditionalized Analysis) as well as an analysis that considers only those items that participants successfully learned in the initial training phase (Conditionalized analysis), as determined by the criterion test that took place after training, but before our Think/No-Think intervention.

### Analyses of unconditionalized data

For the Same Probe test (Table 1), examination of sphericity indicated that it would be appropriate to apply a Greenhouse-Geisser epsilon of .728 to correct the degrees of freedom. Repeated measures ANOVA revealed a significant effect of condition, *F*_(1.46,56.76)_ = 7.81, *p* = .003, *MSE* = .109, partial *η* ^2^ = .167. Planned repeated comparisons revealed that this effect reflected the effects of participants’ poorer recall success (i.e., number of correct cued recalls exclusive of both errors and omissions) in the No-Think condition than in the baseline Study-Only condition, *F*_(1,39)_ = 4.99, *p* = .031, *MSE* = .096. Recall success in the Think condition was significantly greater than in the Study-Only condition, *F*_(1,39)_ = 6.29, *p* = .016, *MSE* = .064. A follow-up paired comparison indicated that recall in the Think condition was significantly superior to the No-Think condition, *F*_(1,39)_ = 10.0, *p* = 0.03, *MSE* = .316.

**Table 1.** Recall success rates in Same Probe and Independent Probe tests.

Test type		Condition	
	Think	No think	Study only
Same Probe	89.7% *(2.2%)*	80.8% *(2.8%)*	85.7% *(2.2%)*
Independent Probe	52.8% *(2.3%)*	44.8% *(1.9%)*	54.0% *(2.0%)*

Note: SEM shown in parentheses.

For the Independent Probe test, examination of sphericity indicated that it would be appropriate to apply a Greenhouse-Geisser epsilon of .863 to correct the degrees of freedom. Repeated measures ANOVA indicated a significant effect of condition, *F*_(2,78)_ = 10.38, *p* < .001, *MSE* = .118, partial *η* ^2^ = .210. Planned comparisons revealed that this effect reflected participants’ poorer recall success in the No-Think condition than in the baseline Study-Only condition, *F*_(1,39)_ = 21.59, *p* < .001, *MSE* = .344. Recall success in the Think (T) condition was not significantly greater than the Study-Only condition, *F*_(1,39)_ = < 1.0. This absence of additional memory strength engendered by the Think procedure has often been observed in prior studies (e.g., Catarino, Küpper, Werner-Seidler, Dalgleish, & Anderson, 2015; Hellerstedt, Johansson, & Anderson, 2016; Küpper, Benoit, Dalgleish, & Anderson, 2014; Streb, Mecklinger, Anderson, Lass-Hennemann, & Michael, 2016). A follow-up paired comparison indicated that recall in the Think condition was significantly superior to the No-Think condition, *F*_(1,39)_ = 9.45, *p* = 0.004, *MSE* = .129.

**Figure 2.**
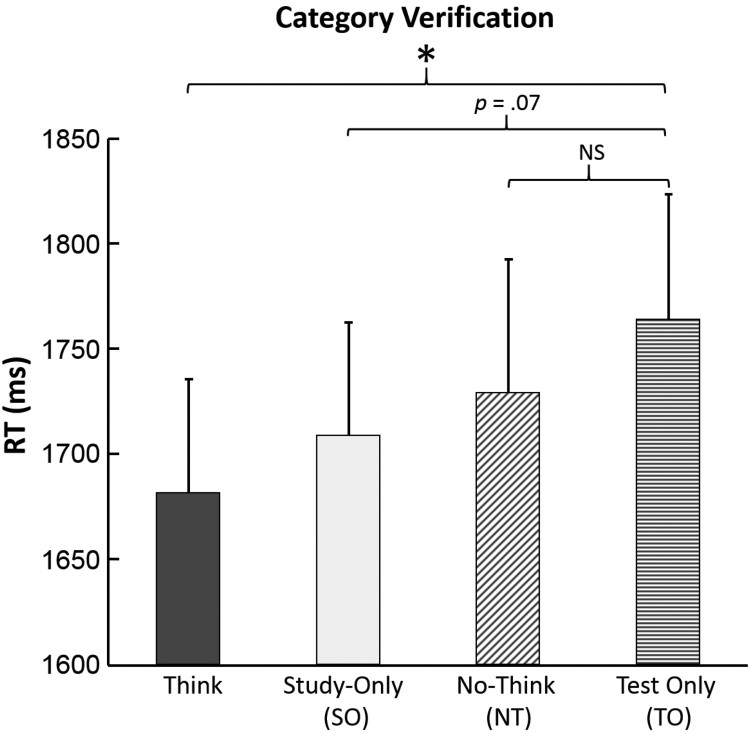
Response time measures for the category verification task. Error bars indicate SEM. *p < .05.

We proceeded to subject the category verification test RT measures (Figure 2) to repeated measures ANOVA, in which four conditions were compared – the three prior conditions and the test-only condition, serving as a baseline condition for priming effects. This indicted a significant effect of condition, *F*_(3,117)_ = 3.01, *p* = .033, *MSE* = 48076, partial *η* ^2^ = .072. Planned comparisons revealed that this effect reflected faster responses compared to the Test-Only baseline in the Think condition, *F*_(1,39)_ = 5.62, *p* = .02, *MSE* = 270413, marginally faster responses compared to the Test-Only baseline in the Study-Only condition, *F*_(1,39)_ = 3.46, *p* = .07, *MSE* = 122333, but no difference in response speed between the Test-Only baseline and the No-Think condition, *F*_(1,39)_ = 1.42, *p* = .24, *MSE* = 48462. The lack of difference between the Test-Only baseline and No-Think conditions suggest that retrieval suppression weakened conceptual priming. Follow-up paired comparisons indicated that RTs in the Think condition were marginally faster than the No-Think condition, *F_(_*_1,39)_ = 3.74, *p* = .061, *MSE* = 44960. Responses in the Study-Only condition did not differ significantly from those of the No-Think condition, *F*_(1,39)_ < 1.0. RTs in the Think and Study-Only condition did not differ significantly, *F*_(1,39)_ < 1.0. Given that our conceptual priming measure constitutes a type of independent probe test, this finding is in line with previous reports that Independent Probe tests almost never exhibit facilitation effects for Think items (Levy & Anderson, 2008). We believe that this reflects the fact that facilitation due to retrieval practice is largely associative, and therefore is not apparent in tests that circumvent the practiced association.

The unconditionalized data, which includes both learned and unlearned items, therefore provides modest support for our hypothesis that suppression reduces conceptual priming. No reliable priming was observed for No-Think items, compared to the Test-Only baseline condition, suggesting that priming had been reduced. In contrast, we found evidence of conceptual priming for Think items and (marginally) for Study-Only baseline items. One ambiguity, however, is that reaction times in the No-Think and Study-Only baseline conditions did not reliably differ from each other, which is predicted if a true reduction in priming occurred. It should be remembered, though, that the unconditionalized analysis is an imperfect test of our prediction because it includes items that were never learned. Next, we report an analysis that considers only those items that were demonstrably learned by participants.

### Analyses of conditionalized data

The preceding analyses reflect performance on the entire corpus of stimuli probed in the three tests administered after the retrieval suppression intervention. However, memory failures for individual stimuli that were not encoded in the first place could dilute the effects of that intervention by including unlearned items that it would not be possible to suppress; thus, many studies have focused exclusively on successfully encoded items (e.g., Anderson et al., 2004). Accordingly, we also conducted conditionalized analyses of post-intervention memory, excluding any items that had not been remembered in the criterion recall test conducted after the initial learning but before the intervention (Table 2). Mean recall success rate in the criterion test was 81.5%, SD = 10.5%.

**Table 2.** Conditionalized recall success rates.

Test type		Condition	
	Think	No think	Study only
Same Probe	95.2% *(1.6%)*	89.0% *(2.5%)*	93.2% *(1.5%)*
Independent Probe	52.7% *(2.4%)*	45.7% *(2.4%)*	54.3% *(2.2%)*

Notes: Recall success rates only for test probes that were recalled in the pre-suppression training criterion test. SEM shown in parentheses.

For the Same Probe test, examination of sphericity indicated that it would be appropriate to apply a Greenhouse-Geisser epsilon of .655 to correct the degrees of freedom. Repeated measures ANOVA revealed a significant effect of condition, *F*_(1.31,51.12)_ = 4.50, *p* = .029, *MSE* = .060, partial *η* ^2^ = .103. Planned comparisons revealed that this effect reflected the effects of participants’ marginally poorer recall success (i.e., number of correct cued recalls exclusive of both errors and omissions) in the No-Think condition than in the baseline Study-Only condition, *F*_(1,39)_ = 3.50, *p* = .069, *MSE* = .068, partial *η* ^2^ = .082. The recall advantage observed in the Think condition was not significantly different from the Study-Only condition, *F*_(1,39)_ = 2.94, *p* = .094, *MSE* = .017, partial *η* ^2^ = .070. A follow-up comparison indicated that recall in the Think condition was superior to the No-Think condition, *F_(1,39)_* *=* 5.52, *p* = 0.24, *MSE* = .076.

For the Independent Probe test, repeated measures ANOVA indicated a significant effect of condition, *F*_(2,78)_ = 6.82, *p* = .002, *MSE* = .085, partial *η* ^2^ = .149. Planned comparisons revealed that this effect reflected the effects of participants’ poorer recall success in the No-Think condition than in the baseline Study-Only condition, *F*_(1,39)_ = 12.17, *p* = .001, *MSE* = .295, partial *η* ^2^ = .238. As for the Same Probe test, in the Independent Probe test the recall advantage observed in the Think condition was not significantly different from the Study-Only condition, *F*_(1,39)_ < 1.0. Follow-up comparison again indicated that recall in the Think condition was significantly superior to the No-Think condition, *F*_(1,39)_ *=* 6.99, *p* = 0.12, *MSE* = .096.

**Figure 3.**
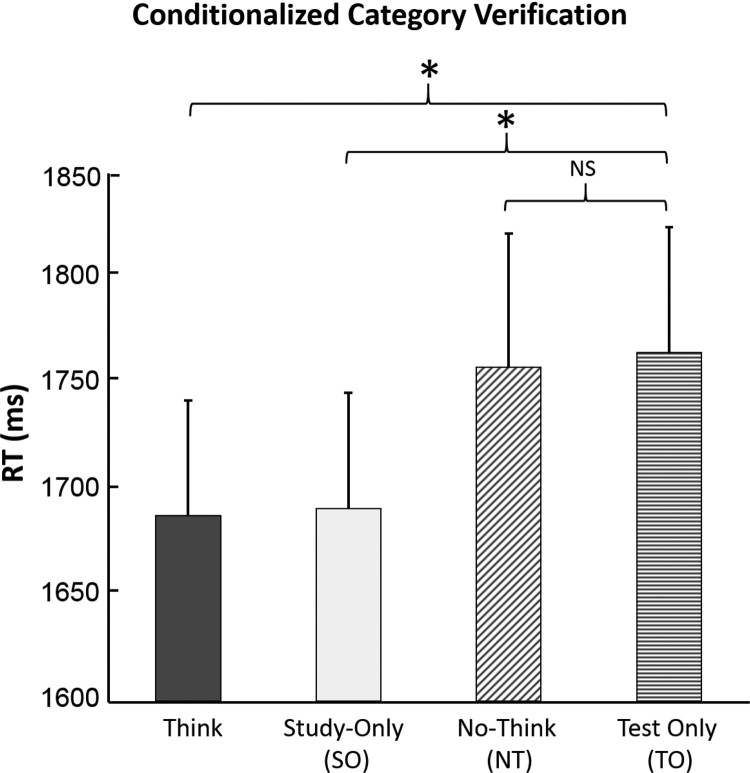
Response time measures for the category verification task, limited to stimuli that were successfully endorsed in the pre-intervention criterion test. Error bars indicate SEM. *p < .05.

We again subjected the category verification test RT measures for the data conditionalized on the words having been successfully recalled in the pre-intervention criterion test (Figure 3) to repeated measures ANOVA, in which four conditions were compared – the three prior conditions and the test-only condition, serving as a baseline condition for priming effects. This indicted a significant effect of condition, *F*_(3,117)_ = 2.83, *p* = .04, *MSE* = 69464, partial *η* ^2^ = .068. Planned comparisons revealed that this effect reflected faster responses compared to Test-Only baseline in the Think condition, *F*_(1,39)_ = 4.61, *p* = .038, *MSE* = 238391, partial *η* ^2^ = .106, and in the Study-Only condition, *F*_(1,39)_ = 5.47, *p* = .025, *MSE* = 17818, partial *η* ^2^ = .123, but not in the No-Think condition, *F*_(1,39)_ < 1.0. Follow-up paired comparisons indicated that RTs in the Think condition were marginally faster than the No-Think condition, *F*_(1,39)_ *=* 3.92, *p* = 0.055, *MSE* = 98386. Furthermore, they indicated that responses in the Study-Only condition were marginally faster than the No-Think condition, *F*_(1,39)_ = 3.43*, p* = 0.071, *MSE* = 89061. The lack of difference between the Test-Only baseline and No-Think conditions, and the slower responding in the No-Think condition than in the T and Study-Only conditions in this conditionalized data, is consistent with the view that retrieval suppression weakens conceptual priming. RTs in the Think and Study-Only condition did not differ significantly, *F* < 1.0, indicating that for conditionalized data as well, explicit associative retrieval does not benefit category verification.

Although our main prediction that suppression would weaken priming in this task received support from the abovementioned data, as with any form of classical null-hypothesis testing, absence of evidence is not evidence of absence. Was priming truly eliminated for No-Think items, as might seem to be the case (Figure 3)? To address this issue, we used Bayes factors to test for evidence of the null hypothesis when comparing reaction times for No-Think and Test-Only items. For this planned comparison, we used a one-sided Bayesian t-test with a Cauchy prior scaled at sqrt(2)/2 (medium scaling). This indicted moderate evidence for priming effects in the Study-Only condition vs. the Test-Only baseline, with a Bayes factor of 3.77; anecdotal evidence for priming effects in the Think condition vs. the Test-Only baseline, with a Bayes factor of 2.6; and crucially, moderate evidence for the null hypothesis that the No-Think condition did not differ from the Test-Only baseline, with a Bayes factor of 0.195. Follow-up analysis conducted with two-sided Bayesian t-tests anecdotally supported the contentions that RTs in the Think condition were faster than in the No-Think condition, with a Bayes factor of 1.92, and that RTs in the Study-Only condition were faster than in the No-Think condition, with a Bayes factor of 1.55. Thus, the conditionalized data provide support for the hypothesis that retrieval suppression mitigates conceptual priming.

## Discussion

In this study, we found that words subjected to retrieval suppression lost the response time priming benefits otherwise observed for studied items in a category verification task. Additionally, we replicated previous findings regarding suppression-induced forgetting effects using both Same Probe and Independent Probe explicit memory tests. The category verification task (CVT) is an indirect memory task making no reference to the encoding episode, and furthermore requires no response production. The demonstration of retrieval suppression effects on this task, alongside the explicit measures of memory employed in most prior research, provides support for the notion that retrieval suppression, at least when performed with direct suppression instructions, affects the actual representations in memory of the suppressed stimuli, rather than being limited to the associative pathways used to retrieve them. More generally, they are consistent with the possibility that retrieval suppression not only affects episodic memory, but also may affect general semantic representations that might support conceptual priming.

One noteworthy aspect of our findings emerged from the procedures employed, in which the CVT was always presented before the Same Probe and Independent Probe explicit tests. Despite the fact that participants were re-exposed to all of the studied items during the CVT, suppression-induced forgetting effects were still found in these later explicit memory tests. Thus, re-exposing the target items did not eliminate the suppression effect on those items, or cause a rebound in accessibility due to any release of inhibition. This finding is notable because it contrasts with studies of list-method directed forgetting, in which it has been found that cueing people, at test, with a portion of the to-be-forgotten study list eliminates directed forgetting for the balance of the list, possibly by reinstating memory strength for the to-be-forgotten items (Bäuml & Samenieh, 2010, 2012). One possible interpretation of the latter finding is that directed forgetting instructions mainly act to shift the participants’ “mental context” away from the first list (Sahakyan & Kelley, 2002), possibly via inhibition of the earlier context (e.g., Anderson, 2005; Bäuml, Hanslmayr, Pastötter, & Klimesch, 2008), making it harder to retrieve the items from that list, even though the items themselves are not inhibited. Re-presenting a portion of the list-one items at test might thus re-instate the context, making the to-be-forgotten items recallable once again. The current failure to find any recovery of suppressed items, despite re-exposure to the entire list, starkly contrast with those reported effects, suggesting that the inhibitory process underlying suppression-induced forgetting affects the items themselves, and not the temporal context (Anderson, 2005). The resilience of the forgetting effect, despite re-exposure, is potentially informative.

The current findings add further evidence that retrieval suppression effects are often cue-independent, consistent with the involvement of inhibitory processes (Anderson & Green, 2001; cf. Anderson & Spellman, 1995). Whether one can infer cue-independence depends, however, on whether the assumptions of the independent probe method are met. A particularly important concern when devising independent probes is that the independent cues are truly unassociated to the original study cues. For example, if independent probes are associated to studied cues, it might lead participants to covertly recall those cues at test, when given independent probes, a process referred to as covert cueing (Anderson, 2003). Some authors have argued that such covert cueing may re-introduce non-inhibitory interference processes that could be mistaken for inhibition (for arguments from retrieval-induced forgetting, see Camp, Pecher, & Schmidt, 2007; Perfect et al., 2004). Although concerns about the independence of independent probes are sometimes warranted (see, e.g., Anderson & Huddleston, 2012), evidence indicates that covert cueing actually reduces or eliminates inhibitory phenomena rather than creating them, consistent instead with the masking hypothesis of covert cueing (Anderson, 2003). For example, on an independent probe test in Retrieval-Induced Forgetting, asking participants to covertly retrieve studied categories eliminated cue-independent forgetting, contradicting predictions of the interference hypotheses and consistent with a compound cueing advantage (Weller, Anderson, Gómez-Ariza, & Bajo, 2013). In the current study, this issue could arise if the Independent Probe cues were associated to the Same Probe cues for the same response item. Although we avoided such associations, some of our stimuli may appear to have a semantic relationship with the original Same Probe cues (see Appendix). However, it should be noted that semantic associations in Hebrew are very different from those in English. Because measures of associative strength in Hebrew are not available for the cues employed, however, we cannot definitively rule out such associations. To the extent that these associations exist, existing data suggests that they should have deflated, rather than caused, cue-independent forgetting, working against our hypothesis (see also Wang et al., 2015, for further evidence against covert interference).

Although the present study, like many previous research projects exploring retrieval suppression, tracks the impact of such suppression on verbal memory, there are reasons to expect that both explicit and implicit effects may generalise to other materials. For example, a variety of other studies (e.g., Catarino et al., 2015; Depue, Curran, & Banich, 2007; Gagnepain, Hulbert, & Anderson, 2017; Küpper et al., 2014) have indicated that retrieval suppression effects may be found for faces, object pictures, and even autobiographical events. Indeed, recent work has suggested that suppression may alter the unintended influence of memories, even when those memories are complex and vivid. For example, Hu, Bergström, Bodenhausen, and Rosenfeld (2015) asked people to engage in a mock crime (stealing a ring), and then do a task that led them to suppress recollections of this real experience. After engaging in the crime, participants were told to pretend that they were being interrogated by police, who were trying to detect evidence of their guilt with an EEG guilty knowledge detection scheme. Participants were presented with cue words associated with the crime while their EEG was recorded; one group was instructed to directly suppress memories of the lab-based crime during this recording session and not to allow it to come to mind during the procedure. Following that intervention, all participants were given an autobiographical Implicit Association Test (IAT). Hu and colleagues reported that the suppression procedure abolished reaction time effects in the IAT that would indicate the presence of guilty knowledge. Additionally, during the suppression procedure, the P300 ERP component in response to crime cues was weaker in the suppression group than in the “guilty” group that did not engage in suppression, and not significantly different from the P300s of participants who had not performed the crime, for whom the cues were not meaningful (cf. Bergström, Anderson, Buda, Simons, & Richardson-Klavehn, 2013). These findings suggest that even complex traces might be suppressible, and that the influence of that suppression can be exhibited on indirect tests, such as the IAT. Based on these findings, we suggest that the present conclusions about conceptual implicit memory may generalise beyond simple verbal materials, although this conclusion must be empirically tested.

Some studies (e.g., Hertel & Calcaterra, 2005; Wang et al., 2015) have noted that interference during retrieval may also be responsible for weakened memories after suppression interventions. However, our demonstration of suppression effects on semantic priming in a category judgment task seems to support an inhibitory account rather than an account that rests solely on retrieval interference. Earlier work on the neural basis of conceptual priming suggests hypotheses about how retrieval suppression may influence conceptual priming. Neuroimaging studies initially identified hippocampal and prefrontal mechanisms underlying the suppression process (Anderson et al., 2004; Depue et al., 2007; reviewed by Anderson & Hanslmayr, 2014). Effective connectivity analyses indicate that the right middle frontal gyrus exerts inhibitory modulation on hippocampal activity to suppress episodic retrieval (Benoit & Anderson, 2012; Benoit, Hulbert, Huddleston, & Anderson, 2014). If priming effects arise outside the hippocampus, however, suppression effects on priming could not be explained by hippocampal modulation. In more recent research, however, Gagnepain et al. (2014) found that when people suppressed retrieval of visual object memories, the dorsolateral prefrontal cortex reduced activation not only in the hippocampus but also in visual cortical regions involved in visual object-perception. Gagnepain et al. (2017) further found that suppressing retrieval of aversive scenes also affects emotional memory traces, and modulates amygdala activity. Based on these observations, these authors argued that retrieval suppression involves the parallel modulation of both the hippocampus and content-specific regions that are reactivated during intrusions of unwanted content. In light of those results, Hu, Bergström, Gagnepain, and Anderson (2017) suggested that conceptual priming could be affected by similar mechanisms, reflecting the action of inhibitory control on neocortical regions involved in conceptual processing. The current results converge with that proposal and suggest that inhibitory modulation of semantic representation regions, possibly in temporal cortex, should accompany modulation of hippocampal activity in the current paradigm.

Thus, the current finding, along with other recent insights into retrieval suppression from behavioural and neuroscience research, may have important implications for approaches to treatment of mental health disorders. Implicit memories can influence behaviour even when the episodic events that prompted their formation are forgotten. People who suffer from unwanted reminders of the past often describe their problematic memory intrusions as vivid, unexpected and uncontrollable. To deal with these intrusive memories, they may utilise strategies of self-distraction or avoidance that are paradoxically associated with more frequent intrusions of negative thoughts, hypervigilance, and negative attributions to intrusions. Because of those memories’ characteristics, there are claims that memory suppression will not help in dealing with such problematic recollection (cited by Hu et al., 2017). We suggest that based on previous findings and on the present results, suppression may indeed influence the implicit activation of unwanted memories. If so, retrieval suppression may, surprisingly, not have the adverse indirect influences on mental health that have often been supposed in clinical research. Rather, the effects noted by clinicians, such as the effects of avoidance and distraction, while real and problematic, may instead often be side effects of not directly engaging with a memory to successfully suppress its retrieval. Alternatively, suppression may be engaged, but deficient in clinical samples, making it harder to achieve good outcomes with suppression when it is attempted (e.g., Catarino et al., 2015). If interventions could be devised to highlight the value of suppression and improve its functioning, we should consider the possibility that they may be of benefit to those with depression (Sacchet et al., 2017) and trauma (Catarino et al., 2015; Hulbert & Anderson, 2018).

## Appendix Appendix

The following list represents rough English translations of the Hebrew words employed as (1) retrieval and category verification targets, (2) the cues used in the original study, suppression practice, and Same Probe trials, and (3) cues used in the Independent Probe trials. All of the original Hebrew terms were single words, and none contained elements found in the cues (e.g., in Hebrew the word for “jellyfish” does not contain “jelly” or “gel”, and the term we used meaning “bookcase” does not contain the word “book”).

The targets are grouped according to the category names, which were used in the category verification task and intended to elicit “yes” responses.

**Table:**

Target	Original cue	Independent Probe cue	Target	Original cue	Independent Probe cue
*Fruit*			*Vegetable*		
fig	cookie	(fig)cake	eggplant	fire	sandwich
kiwi	brown	green	cauliflower	furrow	quiche
grapefruit	grove	citrus	turnip	nerd	root
guyava	seed	tropical	beet	village	red
*Occupation*			*Kitchen utensil*		
clerk	bank	security	blender	storm	whirl
chef	list	restaurant	ladle	sauce	soup
clown	rouge	circus	rolling pin	defense	flour
shoemaker	strap	barefoot	can opener	picnic	lid
*Organ (body part)*			*Farm animal*		
pancreas	toxin	diabetes	goat	hill	beard
shoulder	rash	muscle	pig	odor	sty
womb	life	fetus	chick	nest	egg
stomach	heartburn	digestion	calf	factory	meat
*Sport*			*Raw material*		
rugby	oblong	mud	bronze	judge	medal
fencing	carpet	mask	aluminum	pan	wing
hockey	winter	stick	phosphorous	bomb	sparkler
golf	hole	flag	platinum	bracelet	hair
*Weapon*			*Emotion*		
missile	spark	space	mercy	beggar	teardrop
spear	desert	hunt	hate	rival	attack
javelin	skull	blade	panic	battle	escape
knife	screwdriver	cutting	desire	bachelor	payoff
*Furniture*			*Insect*		
cradle	cat	infant	cricket	summer	garden
bookcase	book	shelf	mosquito	night	itch
mirror	wall	frame	worm	earth	crawl
footstool	old age	lounge chair	moth	garment	lightbulb
Sea creature			Beverage		
whale	strength	fin	cider	blanket	apple
sardine	slenderness	can	lemonade	pleasure	sweet
octopus	shell	arms	tonic	gas	bottle
jellyfish	mucus	gel	mojito	salsa	mint
*Musical instrument*			*Mammal*		
harp	ancient	string	zebra	safari	hoof
harmonica	cowboy	mouth	fox	forest	tail
saxophone	practice	jazz	panther	danger	pink
trombone	army	tube	kangaroo	springboard	pocket
*Clothing*			*Vehicle*		
tie	soiree	groom	tractor	farmer	ploughing
bra	sea	shoulder strap	carriage	park	horse
belt	discipline	skin	taxi	party	receipt
scarf	autumn	neck	electric cart	pension	grandfather
*Room*			*Bird*		
cellar	wine	darkness	peacock	pride	feather
hall	classroom	corridor	raven	wire	black
shelter	bubble	war	swan	water	whiteness
banquet hall	lawn	feast	owl	fable	evening
*Spice*			*Flower*		
cumin	cooked food	felafel	lily	dew	cup
paprika	piquant	powder	sunflower	sun	yellow
cinnamon	song	cake	jasmine	garden	sprig
dill	yoghurt	garlic	lilac	vase	purple

Hebrew equivalents of the following alternative category labels were used in the category verification task in trials querying the above exemplars to elicit “no” responses:

**Table:**

Tool	Prize
Currency	Illness
Colour	Unit of time
Dairy product	Precious stone
Boat	Religion
Office equipment	Music
Unit of distance	Language
Makeup	Sign
Medical instrument	Test
Toy	Medicine
Artwork	Container
